# Exploration of mood spectrum symptoms during a major depressive episode: The impact of contrapolarity—Results from a transdiagnostic cluster analysis on an Italian sample of unipolar and bipolar patients

**DOI:** 10.1192/j.eurpsy.2022.20

**Published:** 2022-05-31

**Authors:** Ludovico Mineo, Alessandro Rodolico, Giorgio Alfredo Spedicato, Andrea Aguglia, Simone Bolognesi, Carmen Concerto, Alessandro Cuomo, Arianna Goracci, Giuseppe Maina, Andrea Fagiolini, Mario Amore, Eugenio Aguglia

**Affiliations:** 1 Department of Clinical and Experimental Medicine, University of Catania, Catania, Italy; 2 Department of Banking and Insurance, Catholic University of Milan, Milan, Italy; 3 Department of Neuroscience, Rehabilitation, Ophthalmology, Genetics, Maternal and Child Health, Section of Psychiatry, University of Genoa, Genoa, Italy; 4 IRCCS Ospedale Policlinico San Martino, Department of Neurosciences, Genoa, Italy; 5 Department of Molecular Medicine, University of Siena, Siena, Italy; 6 Rita Levi Montalcini Department of Neuroscience, University of Turin, University Hospital San Luigi Gonzaga, Turin, Italy

**Keywords:** Mixed depression, Mood spectrum, Subthreshold hypomania, “with mixed features” specifier, cluster analysis

## Abstract

**Background:**

Subthreshold hypomania during a major depressive episode challenges the bipolar-unipolar dichotomy. In our study we employed a cross-diagnostic cluster analysis - to identify distinct subgroups within a cohort of depressed patients.

**Methods:**

A k-means cluster analysis— based on the domain scores of the Mood Spectrum Self-Report (MOODS-SR) questionnaire—was performed on a data set of 300 adults with either bipolar or unipolar depression. After identifying groups, between-clusters comparisons were conducted on MOODS-SR domains and factors and on a set of sociodemographic, clinical and psychometric variables.

**Results:**

Three clusters were identified: one with intermediate depressive and poor manic symptomatology (Mild), one with severe depressive and poor manic symptomatology (Moderate), and a third one with severe depressive and intermediate manic symptomatology (Mixed). Across the clusters, bipolar patients were significantly less represented in the Mild one, while the DSM-5 “Mixed features” specifier did not differentiate the groups. When compared to the other patients, those of Mixed cluster exhibited a stronger association with most of the illness-severity, quality of life, and outcomes measures considered. After performing pairwise comparisons significant differences between “Mixed” and “Moderate” clusters were restricted to: current and disease-onset age, psychotic ideation, suicidal attempts, hospitalization numbers, impulsivity levels and comorbidity for Cluster B personality disorder.

**Conclusions:**

In the present study, a clustering approach based on a spectrum exploration of mood symptomatology led to the identification of three transdiagnostic groups of patients. Consistent with our hypothesis, the magnitude of subthreshold (hypo)manic symptoms was related to a greater clinical severity, regardless of the main categorical diagnosis.

## Introduction

Strong evidence supports the high frequency of contrapolar symptoms in patients suffering from a major depressive episode (MDE) [1–3]. In a recent systematic review, the presence of three or more (hypo)manic symptoms in unipolar and bipolar depression is reported to range from 23 to 35%, respectively [4]. These percentages are significantly increased when a lower number of symptoms is considered [5–7]. Yet, despite its clinical relevance, subthreshold hypomania in patients with an ongoing MDE poses several issues in terms of psychopathological characterization, classification, diagnosis, and treatment [8, 9]. In 2013, the Diagnostic and Statistical Manual for Mental Disorders, Fifth Edition (DSM-5) [10] introduced the “with mixed features specifier” (MFS), applicable to manic, hypomanic and MDEs, both in bipolar disorder (BD) type I and II and in Major Depressive Disorder (MDD). This substantive update was meant to replace the DSM, Fourth Edition, Text Revision (DSM-IV TR) [11] narrow diagnostic category of “Mixed Episode,” providing clinicians with more sensitive criteria, able to address the highly prevalent subsyndromal presentations of mixed states [12, 13]. Furthermore, the fulfillment of the MFS criteria in MDD was expressly indicated by the DSM-5 as a risk factor for the development of BD type I and II, warning clinicians about the need for a clinical evaluation over time, also in the perspective of a potential diagnostic transition. Consequently, the addition of the MFS to MDD was interpreted as a theoretical structural bridge between MDD and BD, positing a more spectrum-oriented approach to mood disorders [14, 15], coherent with the DSM-5 overarching principle of closer integration between the categorical and dimensional model [16].

Nevertheless, this nosologic change was judged to be controversial by several authors and much of the criticism focused on the diagnostic subtype of the MDE “with mixed features”. Indeed, the threshold number of symptoms was deemed arbitrary, as was the choice to retain as mixed features only those manifestations belonging to the manic polarity, and excluding the so-called “overlapping symptoms” such as irritability, psychomotor agitation, and distractibility [17–19]. As remarked by several psychopathologists, the DSM neo-Leonhardian taxonomy of mood disorders, based on polarity (depression and mania as extreme poles of a bipolar dichotomy) rather than on the course and recurrence of the episode, constitutes a theoretical model, per se, unsuitable to offer a diagnostic prototype that would properly target the complexity of mixedness in the real-world clinical setting [20–22].

Starting from a lifetime spectrum approach to mood disorders as opposed to the rigid dichotomic DSM classification category, researchers of the Spectrum Project Collaborative Group developed a self-report tool (Mood Spectrum Self-Report [MOODS-SR]) that is functional for a dimensional model-based evaluation of mood episodes. This tool factorizes affective symptomatology into distinct domains (mood, energy, cognition, and rhythmicity), considering subthreshold-level manifestations of unipolar and bipolar mood psychopathology [23, 24]. Similarly, Malhi et al. proposed the so-called Activity Cognition Emotion (ACE) model, which deconstructs any mood episodes into three main components, describing mixed states as the product of nonsimultaneous changes in these domains [25], reprising the early Kraepelinian classification [26, 27]. Far from being a mere speculative issue, the availability of a valid nosologic framework, accounting for subthreshold hypomania, is fraught with several implications at different levels, including diagnostic recognition, treatment strategy, and research direction [28–30]. Indeed, the unavailability of shared operational criteria has also been a limitation for studies aimed at exploring the neurobiological underpinnings of mixed depression. The vast majority of findings on altered monoaminergic function, hypothalamic–pituitary–adrenal (HPA) axis dysfunction, hyperinflammation, and circadian dysregulation in mixed states are derived from research focused on mixed mania [31]. Therefore, the applicability of the aforementioned pathophysiological mechanisms to mixed depression is purely conjectural.

The present study aimed to identify distinct subgroups using a cross-diagnostic cluster analysis, based on the exploration of mood symptoms, according to a spectrum approach within a cohort of patients admitted with current unipolar and bipolar depression. Cluster analysis is a statistical technique that identifies subgroups as defined by selected features and whose application to heterogeneous and multidimensional disorders, such as MDD, may help to deconstruct disease complexity, contribute to the development and validation of diagnostic criteria, and support tailored treatment plans [32].

After identifying different clusters, we evaluated how cluster membership could be related to diagnostic categories and clinical and psychopathologic factors, hypothesizing that the degree of contrapolar symptomatology may be related to a more severe clinical phenotype of MDE.

## Methods

### Sample

A post hoc cluster analysis was performed on a data set derived from a multicenter cross-sectional study, conducted in three Italian University Hospitals (Siena, Catania, and Turin). The sample consisted of 300 adult individuals with a previously established DSM-5 diagnosis of either MDD or BD. The patients were recruited during their hospital stay, after being informed about the study focus and its voluntary nature. Clear assurance of confidentiality, anonymity, and absence of clinical management implications was also provided. Inclusion criteria were: (a) age >18 years at entry of the study, (b) current diagnosis of a MDE, confirmed by the Mini International Neuropsychiatric Interview (MINI) for DSM IV-TR [11], and (c) ability and willingness to sign a written informed consent. The exclusion criteria comprised a current or past diagnosis of any schizophrenia spectrum disorder, organic psychiatric disorder, major neurocognitive disorder, intellectual disability, or any other neurological condition that may have interfered with the comprehensive evaluation of the patient. It was also required that patients had not received any major pharmacotherapy changes in the last 3 weeks. Each center enrolled 100 patients. The Institutional Review Boards at the Universities of Siena, Catania, and Turin reviewed and approved all the study procedures. The data were collected in compliance with the current version of the Helsinki Declaration and were obtained after written informed consent was received. The complete data set is available from the authors upon request.

### Assessment

A comprehensive psychiatric diagnostic assessment was conducted using the MINI, while sociodemographic and additional clinical characteristics were collected utilizing a semi-structured interview, used in two previously published studies [33, 34]. Patients were also assessed using the Barratt Impulsiveness Scale (BIS-11) [35, 36], the Short Form 12-Item Health Survey (SF-12) [37], the Sheehan Disability scale (SDS) [38], the Clinical Global Impression-Severity scale (CGI-S) [39], and the Seasonal Pattern Questionnaire Assessment (SPAQ) [40].

A dimensional evaluation of the current MDE was carried out by completing the last-month version of the MOODS-Self Report (MOODS-SR), developed from the Structured Clinical Interview for Mood Spectrum (SCI-MOODS) [25]. It is a psychometrically robust questionnaire, specifically structured for a dimensional assessment of mood episode phenomenology. It consists of 161 items, coded as present or absent, for a span of at least 3–5 days over the past month and organized into three depressive and three (hypo)manic domains. MOODS-SR items are targeted at examining energy levels, cognitive features, and affective symptoms, including signs and subthreshold manifestations of mood dysregulation. An adjunctive domain assesses disturbances and rhythmic changes in neurovegetative functions. The MOODS-SR was shown to be reliable with a substantial agreement between the self-report and the interview formats, as expressed by intraclass correlation coefficients (ICC) ranging from 0.88 to 0.97 [24].

The internal structure of MOODS-SR was further divided into six depressive factors (depressive mood, psychomotor retardation, suicidality, drug illness-related depression, psychotic features, and neurovegetative symptoms) and five manic factors (psychomotor activation, mixed instability, spirituality/mysticism/psychoticism, mixed irritability, and euphoria), identified by subsequent factorial analyses studies [41, 42]. The domain and factor scores were obtained as a count of the specific MOODS-SR items endorsed. The scoring procedures are described in detail at www.spectrum-project.org and in the cited papers [41, 42].

### Statistical analyses

Descriptive statistics were reported as frequencies and percentages for categorical variables and as a mean and standard deviation for continuous variables with a normal distribution; nonnormal variables were reported as mean, median, and interquartile range (IQR). For each variable, the normality of the distribution was tested using a Shapiro–Wilk test. A Spearman’s correlation test was used to determine the correlation between the number of depressive and the number of (hypo)manic items in the total sample and the two main diagnostic groups.

In this study, we carried out a *k*-means cluster analysis based on the scores of the six MOODS-SR depressive and (hypo)manic domains.

The optimal number of clusters was determined using the NbClust package [43] implemented in the R software. The NbClust package allows for the comparison of 30 distinct clustering validity indices and recommends the best solution according to a majority rule, that is, the optimal number of clusters is the one supported by the relative majority of the cluster validity indices. The search for the optimal number of clusters was a priori set between one and five, with three being selected as the optimal number of clusters. After the clusters were formed, an initial set of one-way analyses was performed to verify whether the distribution of a group of sociodemographic and clinical variables differed among the clusters. The variables that were tested included: gender, age, age at disorder onset, primary diagnosis (BD vs. MDD), DSM-5-MFS diagnosis, Koukopoulos Mixed Depression (KMxD) diagnosis [44], current psychotic ideation, current suicidal ideation, lifetime hospitalizations, lifetime suicide attempts, comorbidity of any anxiety disorders, substance use disorders, cluster A, cluster B, and cluster C personality disorders, family history of mood disorders, CGI-S score, BIS-11 total score, SDS total score, SPAQ total score, SF-12 Physical Component Summary (SF-12-PCS) score, and SF-12 Mental Component Summary (SF-12-MCS) score. The assessment of DSM-5-MFS and KMxD criteria was carried out through the analysis of clinical records and by using proxy criteria derived from H.D.R.S., Y.M.R.S., and M.I.N.I. items. This reviewing procedure was independently conducted by three trained adult psychiatrists with a substantial experience in the field of mood disorders. The overall mean percentage agreement was 88.50% (range, 82–100%). We also assessed if there were significant intercluster differences in the scores of the internal depressive and (hypo)manic MOODS-SR factors.

The differences between the clusters were verified with suitable one-way analyses (ANOVA, Kruskall Wallis, and chi-square tests), depending on the normal/nonnormal distribution of the variables. If significant intergroup differences were detected, we performed appropriate pairwise post hoc comparisons, adjusted for multiple comparisons (post hoc analysis with Tukey’s adjustment). Finally, a subset of variables (i.e., the ones proven to significantly differ between the Mixed and the Moderate clusters, and also “suicidal ideation”) were modeled as outcomes of generalized linear models (GLMs) (logistic, Poisson or normal, depending on the distribution of the outcome), while the MOODS-SR factors represented the assumed predictors.

The coefficients of the GLM were estimated using elastic-net penalty regularization. The H_2_O R package [45] was used to fit the logistic regression with the elastic-net penalty. The elastic-net technique optimally combines two penalties on the coefficients being estimated, the Least Absolute Shrinkage and Selection Operator (LASSO) (L1) and the Ridge (L2). Both penalties mitigate the impact of nonrelevant or collinear predictors, by shrinking their coefficients toward zero in the estimation process. This provides a more robust and direct identification of relevant variables, compared to the iterated stepwise approach based on classical regression inference. Thus, under the elastic net method, relevant predictors are indicated by an absolute coefficient greater than 0, instead of by a *p*-value under the significance threshold used in the classical inferential approach. Finally, the GLM performance measures, that is, area under the ROC curve (AUC), *R* squared (*R*^2^) and Akaike’s information criterion (AIC) were estimated using 10-fold cross-validation to avoid overfitting, considering the relatively limited sample size.

All statistical analyses were performed using the *R* Statistical software [46] and associated specific *R* packages like Emmeans [47] and DescTools [48]. The H_2_O *R* package [45] was used to fit the logistic regression with the Elastic net penalty. Statistical significance was assessed by using a 5% threshold except for the Elastic net regression analysis.

## Results

### Characteristics of the total sample

The sample consisted of 300 patients of which 155 (51.7%) had a primary diagnosis of MDD while 145 (48.3%) were affected by BD. Females represented 60.7% of the sample while the mean age was 50.1 (14.7). DSM-5 threshold criteria for MFS were met only by 44 subjects (14.7%), while 165 qualified for the KMxD diagnosis. The mean (median) number and {IQR} of the depressive MOOD-SR items endorsed by the patients with MDD and by the patients with BD were 33.65 (36) {18} and 38.28 (40) {17} respectively, whereas, the mean (median) number and {IQR} of the manic MOOD-SR items experienced by unipolar and bipolar patients were 8.61 (6) {12} and 12.76 (11) {12}, respectively.

The Spearman’s rank correlation test showed a weak positive correlation between total depressive and total manic MOODS-SR component scores within the total sample (*p* = 0.292; *p* < 0.001) and also within both main diagnostic groups (MDD: *p* = 0.299; *p* < 0.001; BD: 0.224; *p* < 0.05). The characteristics of the total sample are reported in Table 1.

**Table 1.** Sociodemographic and clinical characteristics of the sample.

Gender (female), *N* (%)	182 (60.7)
Current age, mean ± SD—(median) [IQR]	50.1 ± 14.7—(50) [21]
Years of education, mean ± SD—(median) [IQR]	11.5 ± 4.50—(13) [5.0]
Marital status, *N* (%)	
Single	109 (36.3)
Married	130 (43.4)
Other	61 (20.3)
Occupation, *N* (%)	
Unemployed	118 (39.3)
Student	18 (6.0)
Employed	111 (37.0)
Retired	53 (17.7)
Living status, *N* (%)	
Alone	95 (31.7)
With relatives	205 (68.3)
Primary diagnosis, *N* (%)	
Major depressive disorder	155 (51.7)
Bipolar disorder	145 (48.3)
Mixed depression diagnosis, *N* (%)	
DSM-5 mixed features specifier	44 (14.7)
Koukopoulos mixed depression	165 (55.0)
Lifestyle habits	
Smoker, *N* (%)	127 (42.3)
Daily number of cigarettes, mean ± SD	16.6 ± 9.4
Alcohol consumption, *N* (%)	67 (22.3)
Daily alcohol units, mean ± SD	3.0 ± 2.8
Physically inactive, *N* (%)	189 (63.0)
Young mania rating scale, mean ± SD—(median) [IQR]	4.8 ± 4.1—(4.0) [6.0]
Hamilton depression rating scale, mean ± SD	24.3 ± 4.3
Clinical global impression, mean ± SD—(median) [IQR]	4.8 ± 0.8—(4.0) [1.0]
Shehaan disability scale, mean ± SD—(median) [IQR]	20.96 ± 6.90—(22) [11]
Short form 12 item health survey	
Physical component summary, mean ± SD—(median) [IQR]	42.93 ± 10.70—(40.5) [16.3]
Mental component summary, mean ± SD—(median) [IQR]	25.31 ± 10.15—(23.9) [12.9]

### Cluster analysis

**Figure 1.**
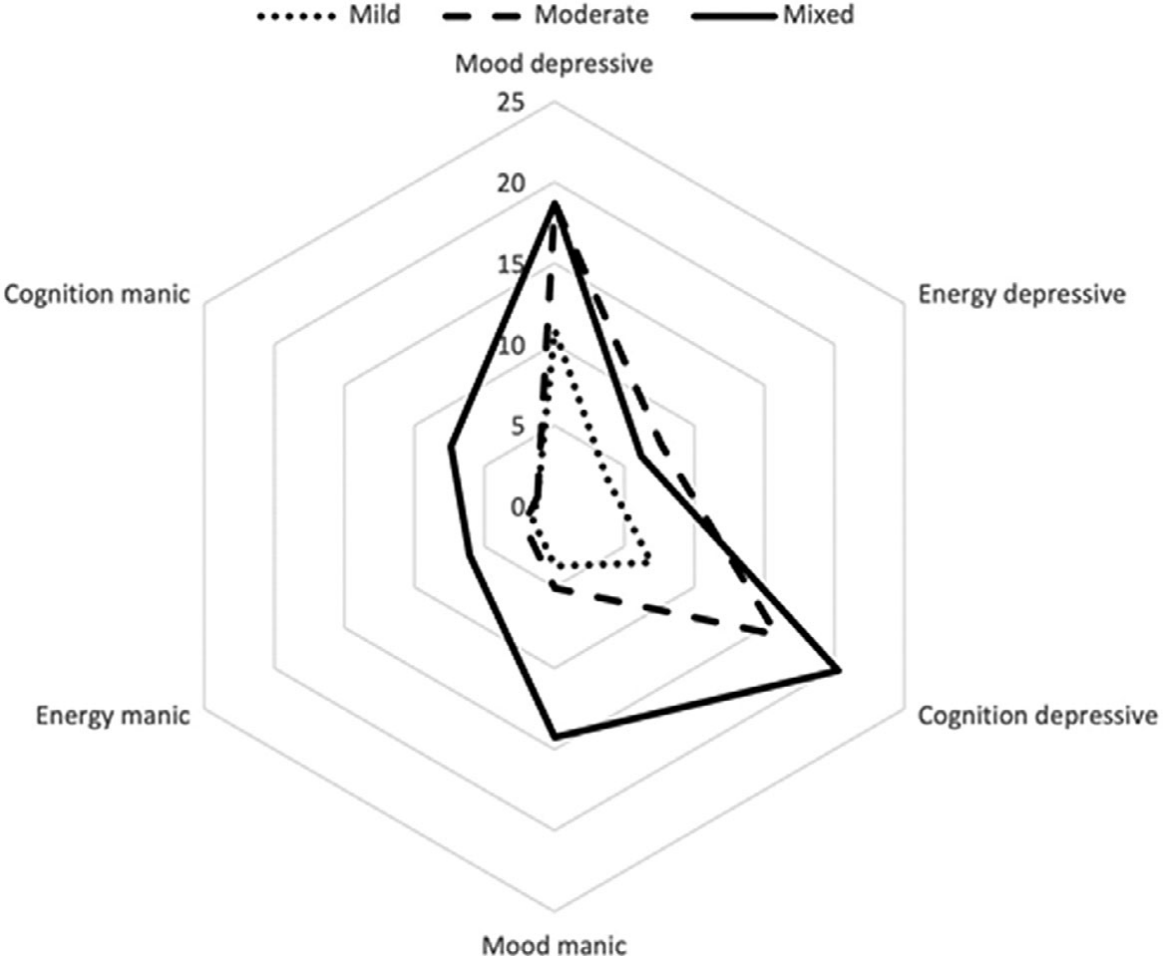
Radar chart representing the distribution of the MOODS-SR domains across the three clusters.

Thirteen out of the 30 validity indices implemented in the NbClust package selected a three-cluster solution, which was therefore adopted as the optimal clustering fit. The number of patients in cluster one (*n* = 98), two (*n* = 158), and three (*n* = 44) accounted for 32.7, 52.7, and 14.6% of the total sample, respectively. After comparing the cumulative scores of the depressive and (hypo)manic MOODS-SR domains for each of the three clusters and the trend of the severity-illness related measures across them, they were labeled as Mild (cluster 1), Moderate (cluster 2), and Mixed (cluster 3) (see Figure 1). Indeed, we were able to detect a group characterized by intermediate levels of depressive symptoms and low levels of (hypo)manic symptoms (Mild cluster), a group with high levels of depressive symptoms and intermediate levels of (hypo)manic symptoms (Mixed cluster) and a large group (Moderate cluster) with depressive and manic symptomatology levels overlapping with those recorded for the Mixed and Mild cluster, respectively.

**Figure 2.**
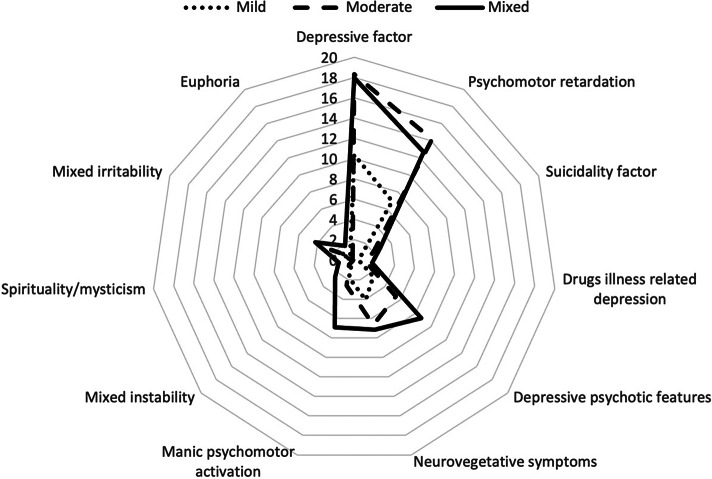
Radar chart representing the distribution of the MOODS-SR factors (according to factor analyses by Cassano) across the three clusters.

A significant main group effect was observed on all scores of the MOODS-SR domains and post hoc tests were run to determine the pairwise differences (see Table 2 for numerical results and Figures 1 and 2 for graphical representations). The Mild cluster was significantly lower than Mixed on all domains and was also significantly lower than Moderate on all domains, except for “cognition manic.” The Mixed cluster was significantly higher than Moderate on all domains, except “mood depressive,” “energy depressive,” and “rhythmicity.” The Mixed and Moderate clusters had similar total average scores in depressive domains, being both significantly higher than Mild. On the other hand, the total average scores of the Mild and Moderate clusters on (hypo)manic domains did not differ significantly but both were significantly lower than Mixed.

**Table 2.** Comparison between the clusters in MOODS-SR domains and MOODS-SR factors.

MOOD-SR domains	Clusters			Intercluster differences	
	*Mild* (*N* = 98)	*Moderate* (*N* = 158)	*Mixed* (*N* = 44)	Overall Kruskal–Wallis *p*-value	Post hoc cluster comparisons
	Mean *(Median)* [IQR]	Mean *(Median)* [IQR]	Mean *(Median)* [IQR]		
Mood depressive	10.87 (11.50)	18.72 (19.00)	18.70 (19.00)	<0.001	Mod. ≃ Mixed > Mild
	[5.75]	[5.00]	[5.00]		
Energy depressive	3.69 (4.00)	7.61 (8.00)	6.20 (6.50)	<0.001	Mod. > Mixed > Mild
	[5.00]	[2.00]	[2.25]		
Cognition depressive	6.93 (7.00)	15.89 (15.00)	20.25 (21.50)	<0.001	Mixed > Mod. > Mild
	[4.00]	[6.00]	[8.25]		
*Depressive symptoms total score*	21.51 (22.00)	42.23 (42.00)	45.16 (46.50)	<0.001	Mod. ≃ Mixed > Mild
	[9.03]	[18.75]	[12.25]		
Mood manic	3.69 (2.00)	5.04 (4.50)	14.27 (14.00)	<0.001	Mixed > Mod. > Mild
	[6.75]	[6.00]	[7.25]		
Energy manic	1.57 (1.00)	2.18 (2.00)	6.04 (6.00)	<0.001	Mixed > Mod. > Mild
	[3.00]	[2.00]	[2.00]		
Cognition manic	1.20 (0.00)	1.20 (1.00)	7.40 (7.00)	<0.001	Mixed > Mod. ≃ Mild
	[2.00]	[2.00]	7.00		
*(Hypo)manic symptoms total score*	6.47 (5.00)	8.43 (8.00)	27.72 (26.00)	<0.001	Mixed > Mod. ≃ Mild
	[10.00]	[9.00]	[10.25]		
Rhythmicity	12.68 (12.50)	16.26 (16.00)	18.38 (19.00)	<0.001	Mod. ≃ Mixed > Mild
	[7.0]	[8.75]	[7.25]		
*Depressive moods-SR factors*					
Depressive factor	10.37 (11.00)	18.35 (18.00)	17.97 (19.00)	<0.001	Mod. ≃ Mixed > Mild
	[5.00]	[5.00]	[5.00]		
Psychomotor retardation	6.91 (7.00)	14.15 (15.00)	12.65 (13.50)	<0.001	Mod. > Mixed > Mild
	[6.00]	[3.00]	[4.00]		
Suicidality factor	0.58 (0.00)	2.12 (2.00)	2.79 (2.00)	<0.001	Mixed ≃ Mod. > Mild
	[0.00]	[4.00]	[4.00]		
Drugs illness related depression	0.49 (0.00)	1.16 (1.00)	1.79 (2.00)	<0.001	Mixed > Mod. > Mild
	[1.00]	[2.00]	[2.00]		
Depressive psychotic features	2.46 (2.0)	5.45 (5.0)	8.73(9.5)	<0.001	Mixed > Mod. > Mild
	[2.0]	[3.0]	[4.25]		
Neurovegetative symptoms	4.07 (4.00)	6.63 (7.00)	7.15 (8.00)	<0.001	Mod. ≃ Mixed > Mild
	[4.00]	[2.19]	[3.00]		
*Manic moods-SR factors*					
Manic psychomotor activation	1.78 (1.00)	2.62 (2.00)	6.90 (7.00)	<0.001	Mixed > Mod. ≃ Mild
	[3.00]	[3.00]	[2.25]		
Mixed instability	0.31 (0.00)	0.66 (0.00)	2.50 (2.00)	<0.001	Mixed > Mod. ≃ Mild
	[0.00]	[1.00]	[3.00]		
Spirituality/mysticism psychoticism	0.08 (0.00)	0.13 (0.00)	1.55 (1.00)	<0.001	Mixed > Mod. ≃ Mild
	[0.00]	[0.00]	[2.00]		
Mixed irritability	1.48 (1.00)	2.50 (2.00)	4.25 (4.00)	<0.001	Mixed > Mod. > Mild
	[2.00]	[1.00]	[1.25]		
Euphoria	0.72 (0.00)	0.32 (0.00)	1.68 (1.50)	<0.001	Mixed > Mild > Mod.
	[1.00]	[0.00]	[2.00]

A significant main group effect was also observed in all scores of the MOODS-SR factors. With regard to (hypo)manic symptomatology, the Mixed cluster reported significantly higher scores in all (hypo)manic factors than both the Moderate and the Mild clusters, while these differed significantly from each other only in “mixed irritability” (Moderate > Mild) and in “euphoria” (Mild > Moderate). Regarding the depressive factors, the Mild cluster showed significantly lower scores for each factor compared to the Mixed and the Moderate clusters, which instead only differed significantly from each other in “psychomotor retardation” (Moderate > Mixed), “suicidality factor” (Mixed > Moderate), “depressive psychotic” features (Mixed > Moderate), and “drugs illness-related depression” (Mixed > Moderate).

### Comparisons among clusters

#### Clinical, diagnostic, and severity variables

There were no gender differences among the three clusters. Significant intercluster differences were found for current age and age at onset of disorder, with post hoc analysis indicating that patients belonging to the Mixed group were significantly younger and had an earlier onset of disease compared to the other two clusters (Mild and Moderate).

Patients with BD were significantly less likely to be present in the Mild cluster than in the Moderate and Mixed clusters. The DSM-5 MFS did not differentiate the three subgroups, unlike the diagnosis of KMxD, which was significantly more prevalent in the Mixed cluster.

Regarding the psychiatric comorbidities, we did not find any significant difference in the prevalence of anxiety disorders across the three subgroups. Conversely, a significantly higher rate of a comorbid cluster B personality disorder, as well as a significantly lower rate of a comorbid cluster C personality disorder among patients belonging to the Mixed cluster, was observed.

When a subset of disease-severity and psychometric variables was considered, significant between-group differences were found for CGI-S, BIS-11, SPAQ, SDS, SF-12 Physical Component Summary (PCS) and SF-12 MCS scores, current psychotic and suicidal ideation, lifetime suicide attempts and the number of hospitalizations, with Mixed cluster patients reporting higher or worse values on each of these measures (except for the SF-12 MCS). Subsequent post hoc pairwise comparisons showed that the Mixed and Mild clusters significantly differed on each of these outcomes. On the other hand, significant differences between the Mixed and Moderate clusters were restricted to BIS-11 total score, current psychotic ideation, lifetime hospitalizations, and suicide attempts. Significant differences between the Mild and Moderate clusters were instead limited to CGI-S scores and current suicidal ideation (see Table 3).

**Table 3.** Comparison between the clusters on clinical characteristics, diagnostic features, and psychometric measures.

	Clusters			Intercluster differences	
	*Mild* (*N* = 98)	*Moderate* (*N* = 158)	*Mixed* (*N* = 44)	Overall	Post hoc cluster comparisons
	*N* (%) *(Median)* [IQR]	*N* (%) *(Median)* [IQR]	*N* (%) *(Median)* [IQR]	p	
Female	60 (61.2%)	97 (61.4%)	25 (56.8%)	0.94	—
Age	49.72 *(49.5)* [14.0]	52.78 *(54.0)* [18.0]	41.02 *(41.0)* [13.75]	**<0.01**	**Mild** ≃ **Mod. > Mixed**
Age at disorder onset	35.50 (25.75)	28.50 (23.75)	22.00 (13.25)	**<0.01**	**Mild** ≃ **Mod. > Mixed**
Bipolar diagnosis	35 (35.7%)	85 (53.8%)	25 (56.8%)	**<0.01**	**Mixed** ≃ **Mod. > Mild**
MFS	15 (15.3%)	18 (11.4%)	11 (25.0%)	0.08	NC
KMxD	42 (46.9%)	85 (53.8%)	34 (77.3%)	**<0.01**	**Mixed > Mod.** ≃ **Mild**
Cluster A pers. dis.	2 (2.0%)	2 (1.3%)	1 (2.3%)	0.8	—
Cluster B pers. dis.	13 (13.3%)	28 (17.7%)	24 (54.5%)	**<0.01**	**Mixed > Mod.** ≃ **Mild**
Cluster C pers. dis.	16 (16.3%)	21 (13.3%)	0 (0.0%)	**0.02**	**Mild.** ≃ **Mod. > Mixed**
Anxiety disorder	28 (28.6%)	37 (23.4%)	10 (22.7%)	0.6	—
Substance use disorder	5 (5.1%)	13 (8.2%)	8 (18.2%)	**0.04**	**Mixed > Mild**
Mood disorders familiarity	45 (45.9%)	87 (55.1%)	25 (56.8%)	0.3	—
CGI-S	4.00 (1.00)	4.00 (1.00)	5.00 (1.00)	**<0.01**	**Mixed** ≃ **Mod > Mild**
Current suicidal ideation	31 (31.6%)	94 (59.5%)	33 (75.0%)	**<0.01**	**Mixed** ≃ **Mod > Mild**
Current psychotic ideation	12 (12.2%)	39 (24.7%)	20 (45.5%)	**<0.01**	**Mixed > Mod.** ≃ **Mild**
Hospitalizations	0.75 *(0.00)* [1.00]	1.77 *(1.00)* [1.00]	2.47 *(2.00)* [2.25]	**<0.01**	**Mixed > Mod. > Mild**
Suicidal attempts	0.13 *(0.0)* [0.0]	0.42 *(0.0)* [1.0]	0.77 *(0.0)* [1.25]	**<0.01**	**Mixed > Mod. > Mild**
BIS-11	60.19 *(61.00)* [13.00]	64.01 *(63.00)* [12.00]	69.34 *(70.00)* [11.00]	**<0.01**	**Mixed > Mod. > Mild**
SPAQ	9.77 *(11.00)* [8.00]	12.46 *(13.00)* [6.75]	12.79 *(13.00)* [4.00]	**<0.01**	**Mixed** ≃ **Mod. > Mild**
SDS	16.96 *(15.00)* [11.75]	22.79 *(24.00)* [6.00]	23.27 *(25.00)* [7.00]	**<0.01**	**Mixed** ≃ **Mod. > Mild**
SF-12 PCS	45.01 *(47.4)* [17.2]	41.42 *(40.1)* [14.5]	37.99 *(36.3)* [12.9]	**<0.01**	**Mild > Mod.** ≃ **Mixed**
SF-12 MCS	31.59 *(30.6)* [12.5]	22.23 *(21.1)* [9.4]	22.43 *(19.9)* [8.9]	**<0.01**	**Mild > Mod.** ≃ **Mixed**

*Note*: The “>”symbol means that the median/mean value of the cluster on the left side of the symbol is statistically different and higher than the cluster on the right side of the symbol, the “≃” symbol means that the median/mean value of the cluster on the left and right sides of the symbol are not statistically different.

Abbreviations: BIS-11, Barratt impulsiveness scale score; KMxD, Koukopoulos mixed depression; MFS, mixed features specifier; SDS, Sheehan disability scale; SF-12 MCS, short form 12 item health survey mental component summary; SF-12 PCS, short form 12 item health survey physical component summary; SPAQ, seasonal pattern questionnaire assessment. Significant differences between groups are reported in bold, cut-off p-value is 0.05.

### Regression analyses

The results of the GLMs are detailed in Table 4. The MOODS-SR factors, identified as positive or negative predictors for the outcomes considered, are indicated by nonzero values for standardized coefficients, with higher values expressing a greater magnitude of influence on the respective outcomes.

**Table 4.** Regression of MOODS-SR Factors with the selected variables.

	Current psychotic ideation	Current suicidal ideation	Lifetime suicide attempts	Lifetime hospitalizations	BIS-11	Cluster B-pers. dis.
MOODS-SR factors	St.coeff.	St.coeff	St.coeff	St.coeff	St.coeff	St.coeff
Depressive factor	**0.19**	**0.05**	0.00	**0.12**	**0.82**	**0.35**
Psychomotor retardation	**0.10**	0.00	0.00	**0.13**	**0.02**	**−0.21**
Suicidality factor	**0.12**	**1.15**	**0.54**	**0.03**	0.00	**0.51**
Drugs illness related depression	0.00	**0.17**	0.00	**0.02**	0.00	**−0.05**
Depressive psychotic features	**0.01**	0.00	0.00	**0.04**	0.00	**−0.34**
Neurovegetative symptoms	**0.01**	0.00	0.00	**0.08**	0.00	**−0.02**
Manic psychomotor activation	**0.32**	**0.18**	**0.22**	**0.06**	**0.63**	**0.25**
Mixed instability	0.00	0.00	**0.12**	**0.07**	**0.61**	**0.29**
Spirituality/Mysticism/psychoticism	**0.12**	0.00	**−0.12**	**0.03**	**0.34**	**0.02**
Mixed irritability	**0.14**	**0.06**	0.00	**0.04**	**2.16**	**0.32**
Euphoria	**0.15**	0.00	0.00	**−0.03**	0.00	**0.34**
*Intercepts*	**−1.29**	**0.24**	**−1.21**	**0.36**	**63.55**	**−1.53**
*GLM type*	Logistic	Logistic	Poisson	Poisson	Gaussian	Logistic
AUC	0.71	0.81				0.78
*R*^2^					28%	
AIC			1139.00	458.00		

*Note: Significant variables in bold.*

Abbreviations: AIC, Akaike’s information criterion; AUC, area under the ROC curve; BIS-11, Barratt impulsiveness scale score; GLM, general linear model; *R*^2^, *R* squared; Stand. coeff., standardized coefficients.

## Discussion

In the present study, we aimed at clustering a sample of inpatients admitted for a MDE in the context of either MDD or BD, based on a spectrum evaluation of mood symptomatology to ascertain whether subthreshold contrapolar symptoms may act as discriminant and moderating severity factors of a current MDE. Before performing the cluster analysis, we checked the relationship between the depressive and (hypo)manic components, finding a similar positive correlation between the number of depressive and manic/hypomanic items, experienced by patients with BD or MDD. This linear relationship had already been found in a previous study by Cassano et al. in a sample that included patients with remitted recurrent unipolar depression and patients with current bipolar depression [49].

Actually, the relationship between depressive and manic symptoms has been investigated by several cross-sectional and longitudinal studies, none of which found support for the core assumption of a robust negative correlation between contrapolar symptoms, posited by the unidimensional model of BD, as no fixed relation pattern was identified [50–52]. Thus, depressive and (hypo)manic symptoms might be conceived as two separate dimensions, independently fluctuating even in their subdomains and this conceptualization would imply an orthogonal, rather than a linear approach to nosology, better encompassing the highly heterogeneous realm of mixed forms [20, 53].

The *K*-mean clustering analysis identified three numerically inhomogeneous transdiagnostic clusters, showing distinct profiles of MOODS-SR domains scores. As expected, BD patients were proportionally more represented in the Mixed cluster compared with the other two, but the post hoc analysis revealed a statistically significant difference in BD diagnosis distribution only between the Mixed and Mild clusters. Considering only the Moderate and Mixed clusters, as they share similar levels of depressive symptomatology and BD prevalence rates, the analysis of the between-groups differences on MOODS-SR factors suggests the presence, in our sample, of two phenotypes of bipolar depression distinguished by different combined degrees of inhibition and hyperactivation. Our findings can be added to those of previous studies, to show evidence for heterogeneity in bipolar depression with the identification of subtypes, based on clinical and psychopathological dimensions rather than nosologic categorization (i.e., BD type I and II) [54–56].

In the present study, we also investigated the pattern of distribution across the clusters of two alternative diagnostic constructs for “Mixed depression.” The prevalence of DSM-5 MFS was higher among the Mixed cluster patients with a percentage of 25%, but no significant mean effect of group was found. This finding may appear in contrast to the results of a recent study involving unipolar and bipolar patients suffering from MDE [57], which identified the clinical presentation with DSM-5 MFS criteria as the second strongest association with the cluster burdened by greater illness severity. Some methodological differences can partially account for this contrast in findings (i.e., different mood symptomatology assessment tools, disparities in sample size, recruitment procedures, interrater reliability levels, care settings, and the heterogeneity of the study population). Consequently, we may surmise that the DSM-5 MFS plays the role of a highly specific marker of mixedness, identifying more dramatic mixed presentations while leaving a large portion of mixed episodes underdiagnosed [5, 57].

Interestingly, the alternative diagnostic construct of mixed depression, proposed by Koukopoulos (KMxD) [44], presented higher prevalence rates than DSM-5 MFS in each of the three clusters and it was found to discriminate the Mixed group from the Mild and Moderate ones after a post hoc analysis. Taken together, these findings appear to be consistent with the arguments questioning the diagnostic validity of the DSM-5-MFS, deemed to be poorly sensitive, and phenomenologically focused on pure manic manifestations but unable to capture the critical excitatory and dysphoric components of mixed depression [58, 59]. These components have instead been incorporated into the KMxD criteria and, accordingly, the scores of the “mixed instability” and “mixed irritability” MOODS-SR subdomains were significantly higher in the Mixed cluster compared to the Mild and Moderate ones.

The study of the distribution across the clusters of the select sociodemographic, psychometric, and clinical variables revealed an overall disease-severity gradient from the Mild to the Mixed cluster. The Mixed cluster exhibited a strong association with most of the illness severity, quality of life, and outcomes measures considered, qualifying as a more severe clinical phenotype, consistent with well-established mixed presentations described in the literature [2, 60, 61]. Compared to the patients in Mild and Moderate clusters, those belonging to the Mixed one were characterized by younger age and an earlier onset of disease, a higher number of hospitalizations and previous suicide attempts, the more likely presence of psychotic and suicidal ideation, greater levels of impulsivity, worse self-reported health and higher disability scores. Furthermore, within the Mixed cluster, we recorded higher comorbidity rates of any cluster B personality disorders or any substance use disorder. However, after post hoc pairwise comparisons between the Moderate and Mixed clusters, both characterized by similar MOODS-SR depressive total scores, statistically significant differences were limited to the number of hospitalizations and suicide attempts, psychotic ideation, comorbidity of cluster B personality disorders, and higher impulsiveness levels.

Finally, the potential correlations between the previously mentioned discriminant variables and the MOODS-SR depressive and hypomanic factors were explored. The regression model for the variable “suicide attempts” revealed that—excluding the intuitive correlation with the “suicidality factor”—the main predictors were represented by two (hypo)manic factors, namely “manic psychomotor activation” and “mixed instability,” consistent with the available evidence on the impact of these domains on the psychopathogenic pathway to suicidal behaviors in mood disorders [62–65]. In particular, as suggested by a comparative assessment of the two separate regression models for suicidal ideation and lifetime suicidal attempts, marked emotional lability and dysphoria may be supposed to exert a critical role in governing the transition from suicidal thought to suicidal acts.

Interestingly, the only negative predictor of lifetime suicide attempts was represented by “spirituality-mysticism-psychoticism,” confirming the religious-spiritual dimension as a protective factor against suicidal attempts [66, 67]. Regarding the predictors for the outcome “lifetime hospitalizations,” contrary to the expectation of overlap with the predictors for suicidal attempts, we observed a slightly greater relevance of MOODS-SR factors belonging to the depressive pole. Specifically, psychomotor retardation may be seen as a symptomatic marker of remarkable importance in guiding clinicians whether to opt for patient’ hospitalizations [68–70]. On the other hand, the level of impulsivity exhibited by our patients was associated with a greater number of positive predictors among the MOODS-SR hypomanic factors. Specifically, the mixed irritability factor presented the highest coefficient, followed by the depressive factor. The presence of subthreshold hypomanic symptoms during an MDE could, therefore, exert a multiplying effect on the proportion of impulsiveness already intrinsic to the depressive episode in both bipolar and unipolar patients [71–73]. Finally, the regression analysis carried out for the variable “comorbid cluster B personality disorder” (represented mainly by a borderline personality disorder—BDP) showed a pattern of positive and negative predictors that appears consistent with the phenomenological characterization of BPD. The significant comorbidity of BPD observed among Mixed cluster patients is not surprising but widely reported in the literature [74–76]. Indeed, the phenomenological and clinical similarities between some mixed episodes and BDP represent critical arguments in the psychopathological debate about the possible inclusion of this personality disorder within the bipolar spectrum [77–79].

This study is subject to several limitations that should be considered when interpreting the results. Firstly, the sample size was not large enough to allow for additional homogeneous subgroups (and therefore to estimate alternative optimal clustering solutions) since sufficient power would have been lost if further trait differences between smaller cluster groups were defined.

Secondly, the MOODS-SR questionnaire inquires only whether the item occurred for at least 3–5 days in the past month, without providing any additional information on the entire duration of occurrence and the intensity of each item. Also, given that the instrument assesses the current and lifetime symptoms, that occurred any time in the last month, there might be a recall bias.

Thirdly, since complete pharmacotherapy data are missing, our findings cannot be adjusted for them. Finally, the multicenter nature of the study may have resulted in differences in the policies adopted for patient’ hospitalizations and the definition-criteria of suicide attempts.

## Conclusion

Using a cluster analysis based on a mood spectrum evaluation, this study identified three transdiagnostic clusters in a sample of acutely depressed patients. In support of our hypothesis, the magnitude of subthreshold (hypo)manic symptoms was related to greater clinical severity, regardless of the main categorical diagnosis. The transdiagnostic composition of each cluster and the orthogonal relationship observed in each group between depressive and manic symptoms, would seem to challenge the unipolar–bipolar dichotomy, supporting the existence of a continuum between the two opposite poles and the consequential need for a dimensional probabilistic approach to mood disorder diagnosis. Furthermore, in line with other studies, our results portray the attempt made by the DSM-5 to provide a reliable nosological framework for intra-MDE hypomania through the introduction of the DSM 5-MFS as unsuccessful, because of the intrinsic limits of that diagnostic category in targeting the whole realm of mixed states. On the other hand, this study represents an attempt at subtyping MDEs based on an in-depth exploration of mood spectrum phenomenology, and challenging the limitations of current categorical systems and polythetic diagnostic criteria. The identification of validated subtypes may aid in improving the classification performance and in guiding therapeutic choices (e.g., the use of antidepressants and the selection of a specific class), allowing a reasonable risk stratification regardless of the diagnostic categorical label. Furthermore, patients clustering based on the deconstruction of affective psychopathology may be functional for research into distinct underlying biological processes and for the subsequent development of personalized treatments [80].

## Data Availability

The raw data supporting the conclusions of this article will be made available by the authors, without undue reservation.
